# Why disability inclusion is essential for trachoma elimination

**Published:** 2020-03-30

**Authors:** KH Martin Kollmann, Sofia Abrahamsson, Tim Jesudason

**Affiliations:** 1 Senior Advisor for Neglected Tropical Diseases: CBM, Nairobi, Kenya.; 2 Policy Advisor – Health: Sightsavers, Haywards Health, UK.; 3 Special Projects and Campaign Partnerships: International Coalition for Trachoma Control, London, UK.

**People who have experienced discrimination associated with disease and disability have a unique voice that takes programmes closer to the communities they are designed to benefit.**

**Figure:**
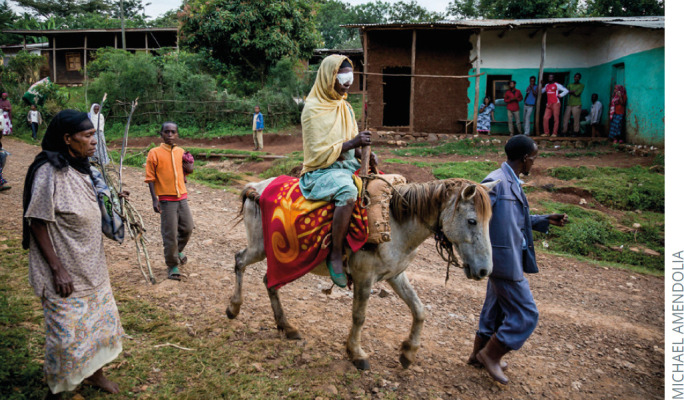
A woman who is blind from trachoma received eyelid surgery to relieve pain from trichiasis. ETHIOPIA

Trachoma, the world's leading infectious cause of blindness, is a cause and consequence of poverty and marginalisation. Communities affected by trachoma often have limited access to good, comprehensive health services and therefore experience high levels of avoidable or correctable complications that result in visual impairment and blindness.

In 2019, the World Health Organization (WHO) released the **World Report on Vision**.^1^ The report states that vast inequities exist in the access to eye health services and that many people with disabilities, including people with vision impairment, are underserved.^1^ To overcome the significant challenges and address access to eye care, the report promotes a people-centred approach, calling for affected people and communities to be central in the design, planning, delivery and evaluation of services.

People with disabilities face a variety of physical and psychosocial barriers to accessing adequate services. In many trachoma-endemic areas, people with disabilities have insufficient access to clean water, toilets and hygiene education,^2^ all of which are needed to reduce the risk of infection with, and transmission of, trachoma. People with disabilities may experience challenges to take part in mass drug administration (MDA) or surgical outreach campaigns without effective support.

The systematic inclusion of people with disabilities and other marginalised groups into trachoma and general eye health programmes is critical to overcome these barriers; however, it will require new thinking and approaches. Programmes must collect data that can be disaggregated by disability during baseline and impact surveys in order to identify the magnitude and pattern of disabilities, inform approaches and identify appropriate partnerships. All of these are needed in order to plan, implement and monitor tailored interventions.

Working in partnership with community-based organisations such as disabled people's organisations (DPOs) will be an important component of this process. By working with DPOs, programmes can develop effective strategies, such as sensitising case-finders ahead of door-to-door screening to identify and treat everyone that needs surgery. For people who are irreversibly blind or visually impaired, programmes must develop effective and comprehensive referral pathways to medical and psychosocial rehabilitation services – including those that support people with livelihood, mental wellbeing and the management of stigma.

The ongoing development of universal health coverage packages at national level provides an opportunity to advocate for the inclusion of people with disabilities in the planning, implementation and monitoring of eye health services. A systematic approach, with specific indicators disaggregated by disability on MDA and surgical services coverage, as well as the accessibility of water, sanitation and hygiene infrastructure, will enhance awareness and assist in ensuring that programmes become fully inclusive. Together, the trachoma, eye health and neglected tropical disease (NTD) communities can support inclusive approaches in health services by collaborating and engaging at national, regional and global levels.

To achieve the 2030 Sustainable Development Goals (SDGs), including Goal 3 on “healthy lives and well-being for all”, we should focus on target 3.3 on communicable diseases including NTDs and 3.8 on universal health coverage. It is essential to fiercely advocate for the participation and inclusion of people with disabilities in addition to other marginalised groups, including women, older people, refugees and indigenous and nomadic communities. There is an increasing body of evidence that participation of communities that are themselves affected makes NTD programmes more effective and increases community ownership and programme sustainability. This can help to secure the great progress already made so far and support the crucial last mile towards achieving elimination of trachoma as a public health problem. Working together with one voice, trachoma, NTD and eye health communities have an opportunity to support and accelerate government efforts to achieve vision for all and truly leave no one behind.

## Recommendations

- Investigate the impact of disability on the accessibility and acceptability of all trachoma interventions and adapt programmes accordingly
- Address the impact of trachoma on disability-related stigma and mental wellbeing
- Collect, analyse and use disability-disaggregated data and conduct implementation research on inclusive preferred practises
- Promote intersectoral working that promotes the formation and inclusion of disabled people's organisations (DPOs)
- Promote human rights-based participatory and people-centred approaches.
